# Supplementary material for: Products of the Parkinson's disease-related glyoxalase DJ-1, D-lactate and glycolate, support mitochondrial membrane potential and neuronal survival
Source: Biol Open. 2014 Jul 25;3(8):777–84. doi: 10.1242/bio.20149399 (PMC4133730; doi:10.1242/bio.20149399)
Supplement: Supplementary Material [file supp_3_8_777__index.html]

Products of the Parkinson's disease-related glyoxalase DJ-1, D-lactate and glycolate, support mitochondrial membrane potential and neuronal survival — Products of the Parkinson's disease-related glyoxalase DJ-1, D-lactate and glycolate, support mitochondrial membrane potential and neuronal survival — Products of the Parkinson's disease-related glyoxalase DJ-1, D-lactate and glycolate, support mitochondrial membrane potential and neuronal survival — Supplementary Material 

# Products of the Parkinson's disease-related glyoxalase DJ-1, D-lactate and glycolate, support mitochondrial membrane potential and neuronal survival

## bio.20149399 Supplementary Material

**Files in this Data Supplement:**

- Supplementary Material - Yusuke Toyoda et al. doi: 10.1242/bio.20149399
