# Supplementary material for: Products of the Parkinson's disease-related glyoxalase DJ-1, D-lactate and glycolate, support mitochondrial membrane potential and neuronal survival
Source: Biol Open. 2014 Jul 25;3(8):777–84. doi: 10.1242/bio.20149399 (PMC4133730; doi:10.1242/bio.20149399)
Supplement: Supplementary Material [file supp_bio.20149399_bio.20149399-s1.pdf]

Biology Open (2014) 000, 1–8 doi:10.1242/bio.20149399

Supplementary Material
Yusuke Toyoda et al. doi: 10.1242/bio.20149399

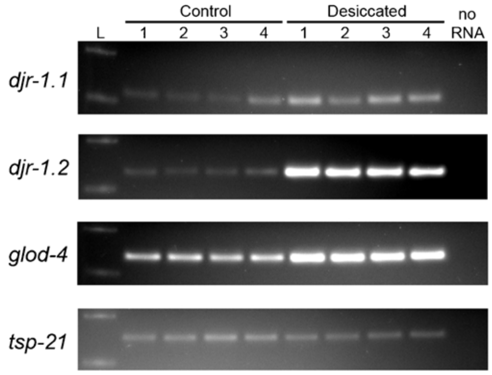

Fig. S1. Upregulation of glyoxalase genes upon desiccation of *C. elegans* dauer. The differential expression of *djr-1.1*, *djr-1.2* and *glod-4* was tested by RT-PCR in four replicates. See Fig. 1A for the procedure. *tsp-21* was a control whose expression did not change by desiccation stress.

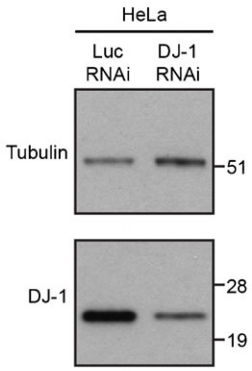

Fig. S2. DJ-1 downregulation by esiRNA. Expression of DJ-1 protein in esiRNA-transfected HeLa cells. DJ-1 and tubulin proteins in the whole lysate were detected by immunoblotting. By a densitometric analysis, RNAi of DJ-1 downregulated its expression by 75% in HeLa cells.

Biology Open (2014) 000, 1–8 doi:10.1242/bio.20149399

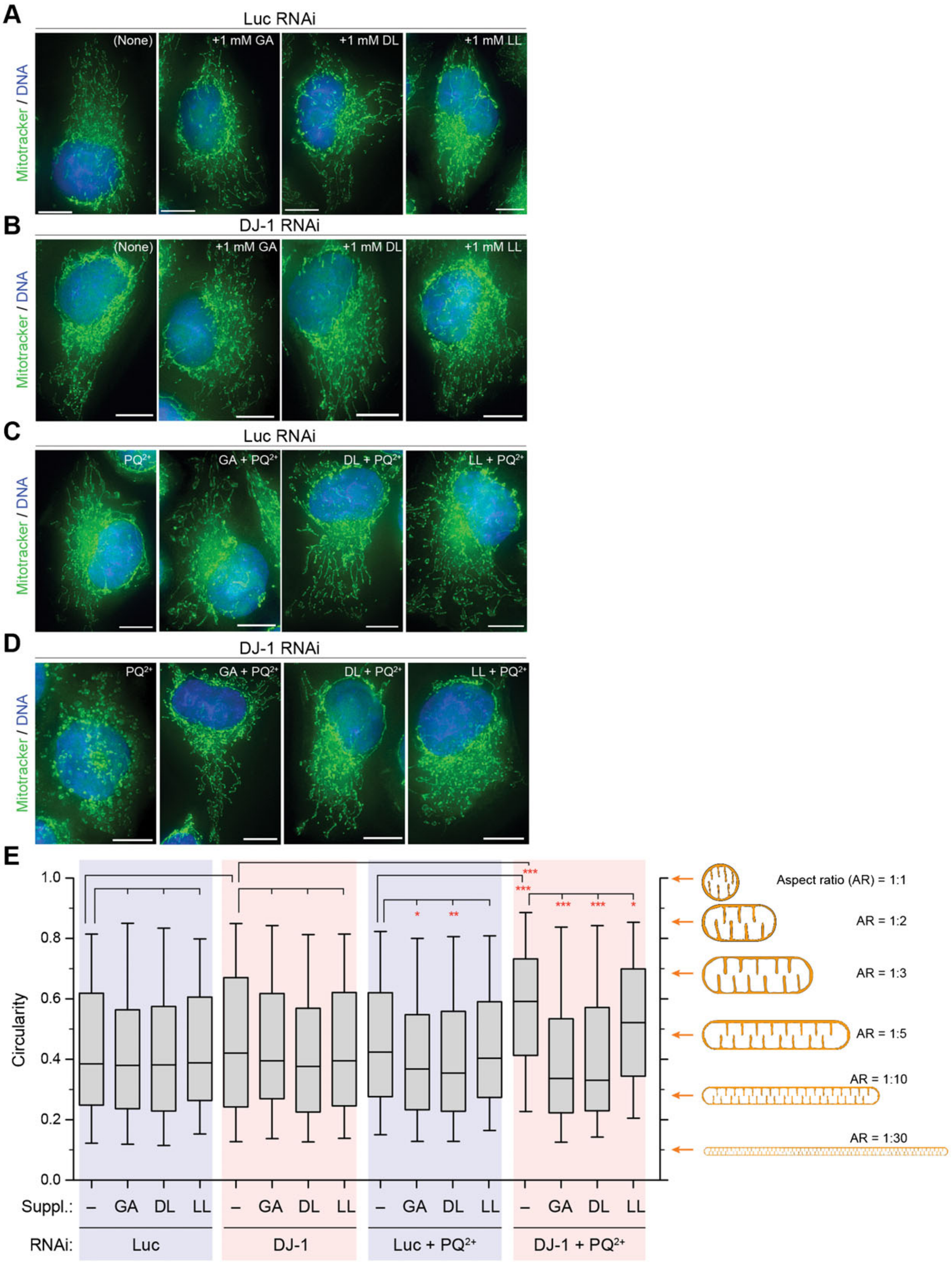

Fig. S3. Glycolate and D-lactate rescue mitochondria structure of paraquat-treated DJ-1 RNAi HeLa cells. (A–D) Mitochondria stained with MitoTracker (green) and DNA (blue) of cells treated with control Luciferase RNAi (A), DJ-1 RNAi (B), control RNAi and paraquat (PQ^{2+}) (C), and DJ-1 RNAi and PQ^{2+} (D). Scale bars: 10 µm. (E) Quantification of the mitochondrial network structure. Circularity of mitochondria in cell periphery was calculated ($n \geq 280$ for each box). On the right, the relation between the mitochondrial shape and circularity is drawn. Circularity in each condition was compared to its own control by one-way ANOVA followed by Tukey's HSD test. *$p<0.05$; **$p<0.01$; ***$p<0.001$.

Biology Open (2014) 000, 1–8 doi:10.1242/bio.20149399

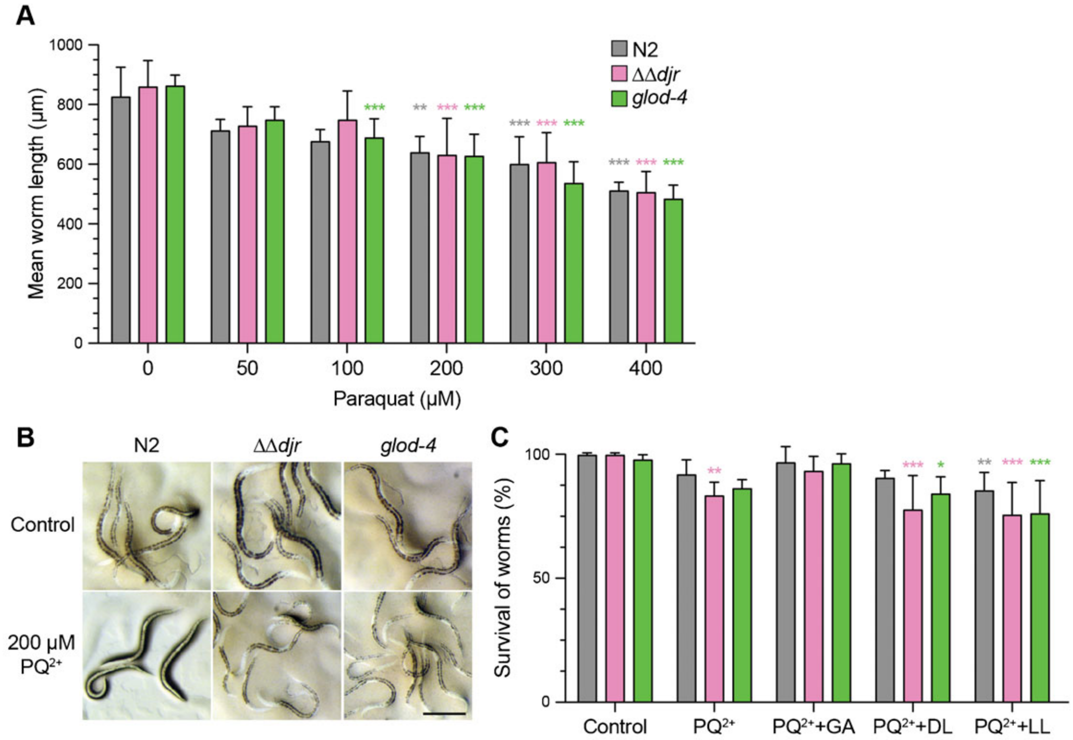

Fig. S4. Effects of paraquat on worm larvae. (A) Length of the worms treated with paraquat (PQ^{2+}). Bars and error bars show the mean and SD, respectively. Sensitivity to PQ^{2+} was comparable between strains ($F=2.334$, $df=2$, $p=0.1$) but overall increased by concentration ($F=81.159$, $df=5$, $p<0.001$). Every strain was compared to its control at different PQ^{2+} concentrations by two-way ANOVA followed by Tukey's honestly significant differences (HSD) test.
(B) Worm larvae treated with PQ^{2+} or control. Scale bar: 250 μm. (C) Survival of the worm larvae treated with 200 μM paraquat and 1 mM of the indicated supplements. Bars and error bars show the mean and SD, respectively. Every strain was affected differently upon each treatment (strain level $F=10.748$, $df=2$, $p<0.001$; treatment level $F=24.467$, $df=5$, $p<0.001$). PQ^{2+} decreased viability of *ΔΔdjr* mutant, which was restored by glycolate (GA), but not by D-lactate (DL), L-lactate (LL). Viability of *glod-4* was not affected by PQ^{2+} significantly; however, the lethality was rescued in a similar way as *ΔΔdjr*. Every strain was compared to its own control by two-way ANOVA followed by Tukey's HSD test. Data were normalized by Freeman–Tukey's double arcsine transformation prior to ANOVA. $^{*}p<0.05$; $^{**}p<0.01$; $^{***}p<0.001$.

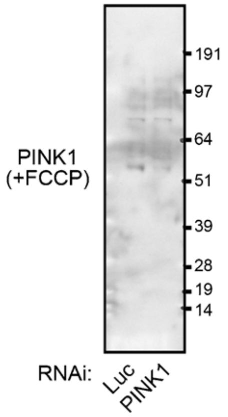

Fig. S5. PINK1 downregulation by esiRNA. Expression of PINK1 protein in esiRNA-transfected HeLa cells. Before harvest, cells were incubated with 10 μM FCCP (Carbonyl cyanide 4-(trifluoromethoxy)phenylhydrazone) for 1 hour. Loading of same amounts of total proteins was confirmed by Ponceau S staining (not shown). A 54 kDa band near to the expected molecular weight (63 kDa) was diminished in the PINK1 RNAi sample.

Biology Open (2014) 000, 1–8 doi:10.1242/bio.20149399

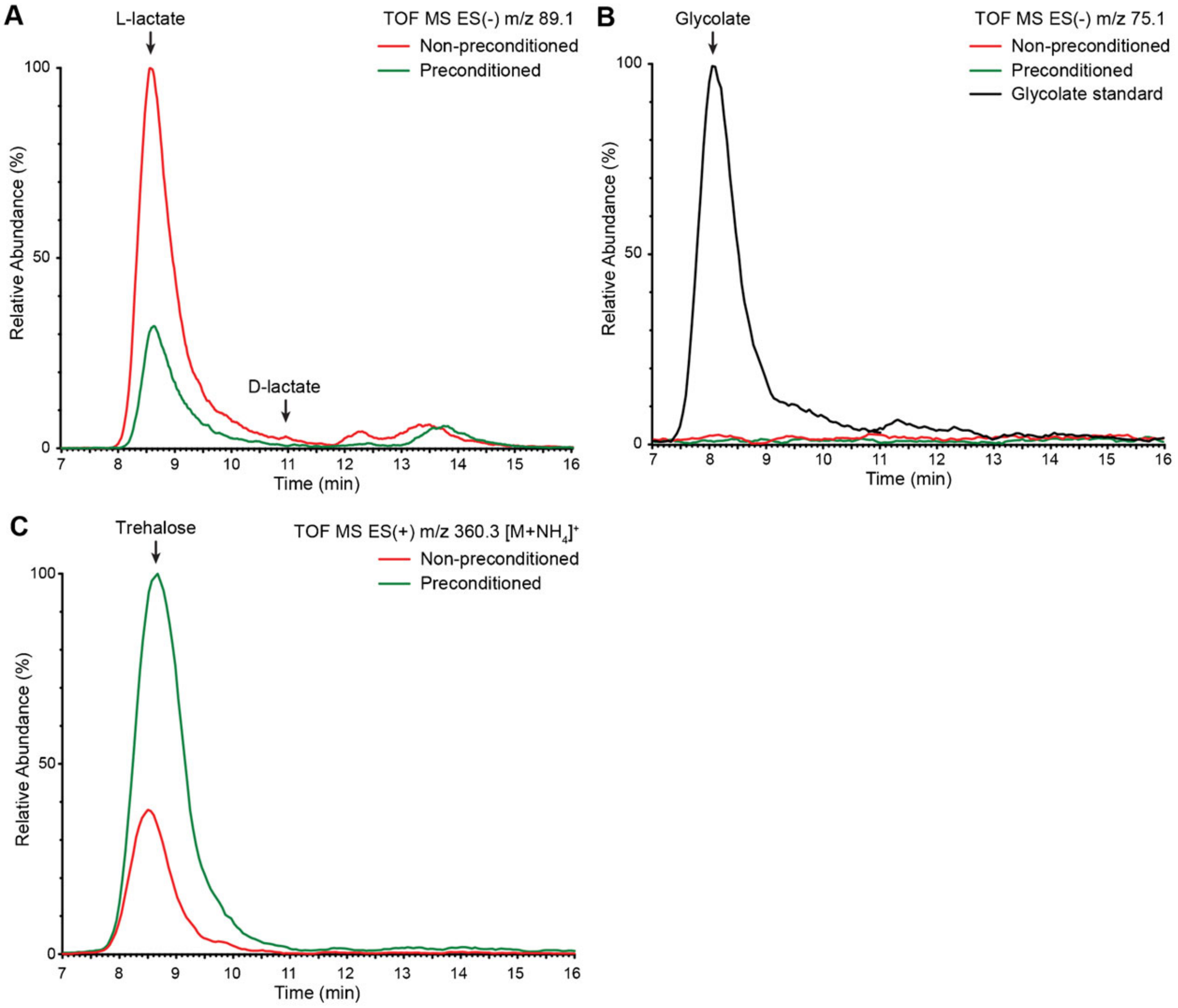

Fig. S6. Detection of α-hydroxy acids and trehalose in worms before and after preconditioning. Selected ion monitoring (SIM) chromatograms for molecules of interest are overlaid. (A) Separation of lactic acid stereoisoforms. L-lactate decreases 3-fold upon preconditioning whereas D-lactate is in trace amount in worms and its abundance is not affected by desiccation stress. (B) No glycolate is detected in worms both before and after preconditioning. (C) Trehalose level is increased more than 2-fold upon preconditioning.

Biology Open

Biology Open (2014) 000, 1–8 doi:10.1242/bio.20149399

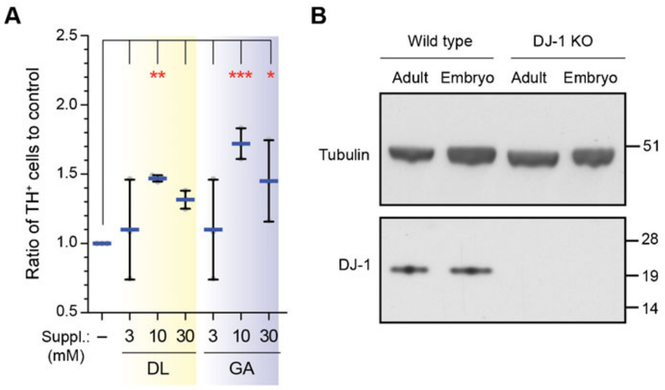

Fig. S7. Glycolate and D-lactate support *in vitro* survival of the dopaminergic neuron. (A) Survival of the primary dopaminergic neurons in the presence of the different concentrations of D-lactate (DL) and glycolate (GA). The primary neurons from wild-type mouse embryos were cultured with the indicated substances for 6 days, fixed, and stained for tyrosine hydroxylase (TH), a dopaminergic neuron-specific marker. The relative number of the TH^+ cells to none-treated control was plotted, with mean (blue) and SD. Each dot indicates an independent experiment. *$p<0.05$; **$p<0.01$; ***$p<0.001$. (B) Immunoblot of DJ-1 in wild-type and DJ-1 mutant mouse brains. Total brains isolated from wild-type and DJ-1 mutant adults and embryos were lysed, and tested for DJ-1 expression. Tubulin is the loading control.

Table S1. Sequences of the primers used in genotyping and RT-PCR

Gene/allele	Primer name	Primer sequence
**Genotyping**		
djr-1.1(tm918)	tm918_ext_fwd	CGACGAGTTGCGTATGAGAA
	tm918_ext_rev	CACAAGTTTTTCGGGGAGAA
	tm918_int_fwd	TATGCCGGATTAGATGGAGC
djr-1.2(tm951)	tm951_ext_fwd	GATTTCTTCGGCGTCTTCTG
	tm951_ext_rev	CACATCTCGGGCCACTATTT
	tm951_int_fwd	AAAATGCAACGACCGACTTC
glod-4(tm1266)	tm1266_ext_fwd	TCCTCCGCTCGCTTTTTCTC
	tm1266_ext_rev	TTGCAAGTTGCTTCGCATCC
	tm1266_int_fwd	TCGAAGCTTTGGTCGTTTCG
**RT-PCR**		
djr-1.1	djr-1.1_fwd	GCCGAAGGAGCTGAGGAAATG
	djr-1.1_rev	AGCACATTTTACCGGTTCGGC
djr-1.2	djr-1.2_fwd	TGAACCTGTCAAATGTGCCAAAGG
	djr-1.2_rev	GGCACTCTGCCAGTTTGCTAC
glod-4	glod-4_fwd	CCTGAAGATAAGCTCGAATCTCTCC
	glod-4_rev	ATGCTCATCTGGATCGGCAAG
tsp-21	tsp-21_fwd	ACAGAGAGAGCTCCAATGCTGC
	tsp-21_rev	TTTCCCACAGTTTTCTGTGCCG
